# Organ Culture Model of Aortic Valve Calcification

**DOI:** 10.3389/fcvm.2021.734692

**Published:** 2021-10-01

**Authors:** Adrian H. Chester, Padmini Sarathchandra, Ann McCormack, Magdi H. Yacoub

**Affiliations:** ^1^Heart Science Centre, Magdi Yacoub Institute, Harefield, United Kingdom; ^2^National Heart & Lung Institute, Imperial College, Imperial College London, London, United Kingdom

**Keywords:** aortic valve, calcification, porcine, adenosine, lipopolysaccharide, osteoblasts, valve interstitial cells, valve calcification model

## Abstract

A significant amount of knowledge has been gained with the use of cell-based assays to elucidate the mechanisms that mediate heart valve calcification. However, cells used in these studies lack their association with the extra-cellular matrix or the influence of other cellular components of valve leaflets. We have developed a model of calcification using intact porcine valve leaflets, that relies upon a biological stimulus to drive the formation of calcified nodules within the valve leaflets. Alizarin Red positive regions were formed in response to lipopolysaccharide and inorganic phosphate, which could be quantified when viewed under polarized light. Point analysis and elemental mapping analysis of electron microscope images confirmed the presence of nodules containing calcium and phosphorus. Immunohistochemical staining showed that the development of these calcified regions corresponded with the expression of RUNX2, osteocalcin, NF-kB and the apoptosis marker caspase 3. The formation of calcified nodules and the expression of bone markers were both inhibited by adenosine in a concentration-dependent manner, illustrating that the model is amenable to pharmacological manipulation. This organ culture model offers an increased level of tissue complexity in which to study the mechanisms that are involved in heart valve calcification.

## Introduction

The development of a medical strategy to treat aortic stenosis is required to alleviate the burden of the increasing numbers of patients who require aortic valve replacement, which is predicted to increase with an aging population (1). The use of pharmacological agents to delay or even remove the need for surgical replacement of diseased valves, or percutaneous implantation, would represent a breakthrough in the treatment of patients with calcific aortic valve disease (2). The translation of information gained from *in vitro* experiments using isolated cells into clinical studies has been hampered by the limitations of available animal models of calcific valve disease (3). This is illustrated by the disappointing results from clinical trials with statins (4, 5), despite data from *in vitro* and *in vivo* studies that supported beneficial effects of statins against the development of valve calcification (6–8).

The use of pathological specimens from humans and cultured cells from humans, sheep and pigs has shed light on the biological mechanisms that mediate the differentiation of valve interstitial cells (VIC) into an osteogenic cell phenotype, which is believed to be the responsible cell phenotype in the development of calcified lesions in the fibrosa layer of the valve (9). Cell-based studies have relied on a variety of endpoints to assess pro-calcifying stimuli, including the expression of osteogenic markers, evidence of osteogenic signaling and the measurement of calcium using calcium specific dyes, radiolabeled calcium or histochemical staining (Alizarin Red and von Kossa) (10–12). With regards to mineralisation, previous studies have questioned the “calcified nodule” model of valve calcification using cultured porcine VIC, whereby analysis of nodules formed in response to TGF-b1 and osteogenic media, which stained positive for Alizarin Red, failed to show the presence of mineralisation using live-cell imaging micro-Raman spectroscopy (13). While the use of human cells is informative, attempts to standardize conditions to induce calcification have shown the dependence on passage number in the response of osteogenic media containing organic phosphate (14). There is also an influence of the compliance of the substrate on which cells are grown with regards to calcification potential (15).

Based on the experience with vascular calcification (16, 17), and knowledge gained with use of cultured cells to induce calcification with respect to the use of lipopolysaccharide (LPS) and inorganic phosphate (12, 18–20), we set out to establish an organ culture model of valve calcification using intact porcine aortic valve leaflets. Such a model would allow assessment of the response of the VIC while they are in their physiological environment with respect to the 3-D architecture of the extracellular environment and the proximity of valve endothelial cells (VEC).

## Methods

### Stimulation of Leaflet Calcification

Porcine hearts were obtained from a local abattoir. Under sterile conditions the aortic valve leaflets were removed from the aortic root and placed immediately in DMEM. Each leaflet was pinned to the base of a 6-well tissue culture plate coated with the silicone elastomer, Sylgard (Sigma) and covered with 3 mL of low glucose DMEM with 0.4% FBS and antibiotics. To induce a calcification response valve leaflets were incubated with LPS and inorganic phosphate (sodium phosphate), with the media being changed every 2–3 days. Experiments were conducted to determine the optimal concentrations of LPS and phosphate, the duration of incubation required, and the variability between different leaflets and within different regions of each leaflet. The experiments were performed in the presence of 0.4 unit/mL pyrophosphatase (Sigma), which reduces levels of pyrophosphate, an endogenous inhibitor of calcification and has been shown to increase the calcifying effect of phosphate in porcine valve cusps (12). Once the conditions for inducing and quantifying a calcification response in the valve leaflets was achieved, the inhibitory effect of increasing concentrations of adenosine was assessed.

### Assessment of Leaflet Calcification

#### Quantification of Calcium Deposition

**Figure 1:**
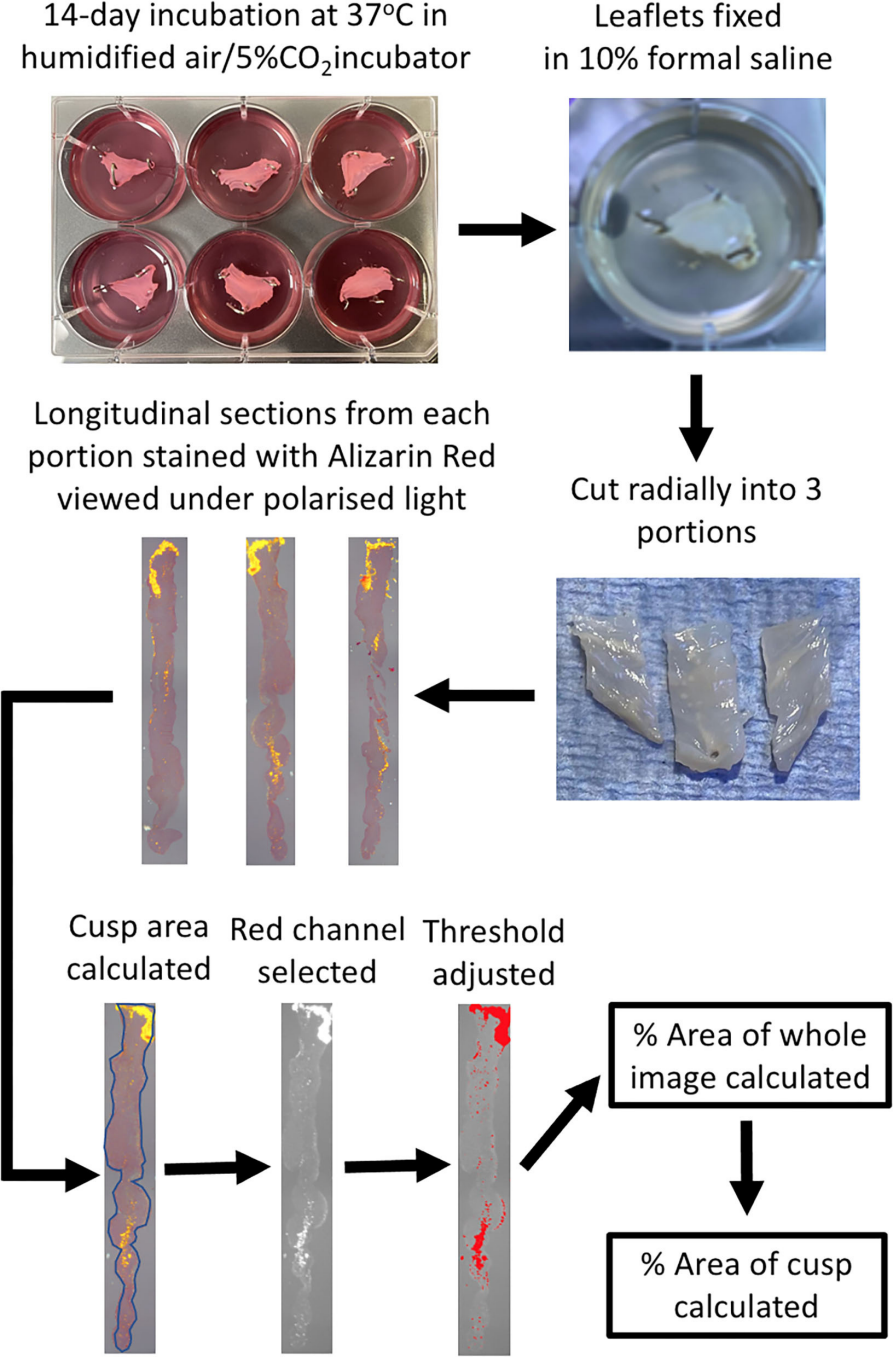
Schematic diagram of the experimental protocol. Following incubation of the pinned-out valve leaflets, they are fixed while still in the wells with 10% formal saline. Each leaflet is cut into 3 portions radially, processed and embedded in wax blocks and cut longitudinal sections cut on a microtome. Alizarin Red staining of a slide from each of the three leaflet portions is viewed under polarized light and then quantified using Image J, to ultimately calculate the percentage of the area of the leaflet that stains for Alizarin Red.

On completion of the experiment, valve leaflets were fixed in 10% formal saline. Valve leaflets were cut into 3 pieces radially (left, center and right portions of the leaflet) and were processed, mounted in wax blocks sectioned and stained with Alizarin Red. Images were taken under polarized light and image analysis was carried out using Image J (Figure 1).

#### Immunohistochemistry for Calcification Markers

Prior to immunoperoxidase staining, 5 μm thick paraffin wax sections of valve leaflets were dewaxed and rehydrated in water. Antigen retrieval was carried out by immersing the slides in 0.1 M citrate buffer (pH 6) and microwaving for 10 min, the slides were then left in the same buffer for a further 20 min followed by tap water wash. Endogenous peroxidases in the tissue were blocked by incubating with 3% hydrogen peroxide for 5 min. To reduce non-specific binding, the slides were incubated with blocking buffer [3% Bovine serum albumin (BSA, Sigma) + 3% normal horse serum (Vector laboratories)], for 30 min. Slides were then incubated overnight in a moist chamber with antibodies against rabbit polyclonal RUNX2 at 1:200 (Abcam), mouse monoclonal Osteocalcin 1:600 (Abcam), rabbit polyclonal Osteopontin 1:500 (Chemicon), mouse monoclonal NF-kB 1:800 (BD transduction), Rabbit monoclonal cleaved caspase 3 1:200 (R&D systems). Antibody dilutions were made in half-strength blocking buffer. Negative controls consisted of blocking buffer. After thorough washing, all the slides were incubated with VECTASTAIN® Elite® ABC-Peroxidase Kit (R.T.U. Universal) for 30 min for each secondary and tertiary antibody (Vector Laboratories). Specimens were then incubated with DAB (Sigma) for 5 min washed well in tap water, stained the nuclei with Haematoxylin for 1 min and mounted using Aquatex (VWR). Stained slides were scanned using Hamamatsu Nanozoomer.

#### Elemental Analysis by Energy-Dispersive X-Ray Spectroscopy

10 μm thick paraffin wax sections were dewaxed and rehydrated to water, air-dried, mounted on SEM stubs coated with Gold/Palladium. Elemental analysis was performed by Energy-dispersive X-ray spectroscopy (EDAX) on a JEOL 6,010 analytical scanning electron microscope.

### Data Analysis

Photomicrographs of the three sections (left, middle and right) from each leaflet, stained with Alizarin Red and viewed under polarized light, or antibody staining viewed under normal light, were analyzed with Image J. Firstly the area of the leaflet was calculated by tracing around the edge of the section (S_Area_). The section was then viewed under the Red channel and the threshold adjusted to highlight only areas of positive staining. The area fraction (AF) for the whole field of view was then calculated (FofV_Area_), which was subsequently used to calculate the percentage area of positive staining of the section [(AF x FofV_Area_/S_Area_) x100]. The mean of the values for three regions of each leaflet was then calculated to give the result for each leaflet. For quantification of antibody staining in **Figure 8**, Image J software was used to calculate the percentage of area stained of photomicrographs of identical size at x40 magnification from regions of whole leaflet sections that were either Alizarin Red positive or negative. Statistical analysis was performed using ANOVA followed by the Dunnett's *post-hoc* test or a *T*-test where appropriate. Numerical data is expressed as the mean ± standard error of the mean. Values for N referred to the number of individual cusps that were studied.

## Results

### Stimulation of Leaflet Calcification

**Figure 2:**
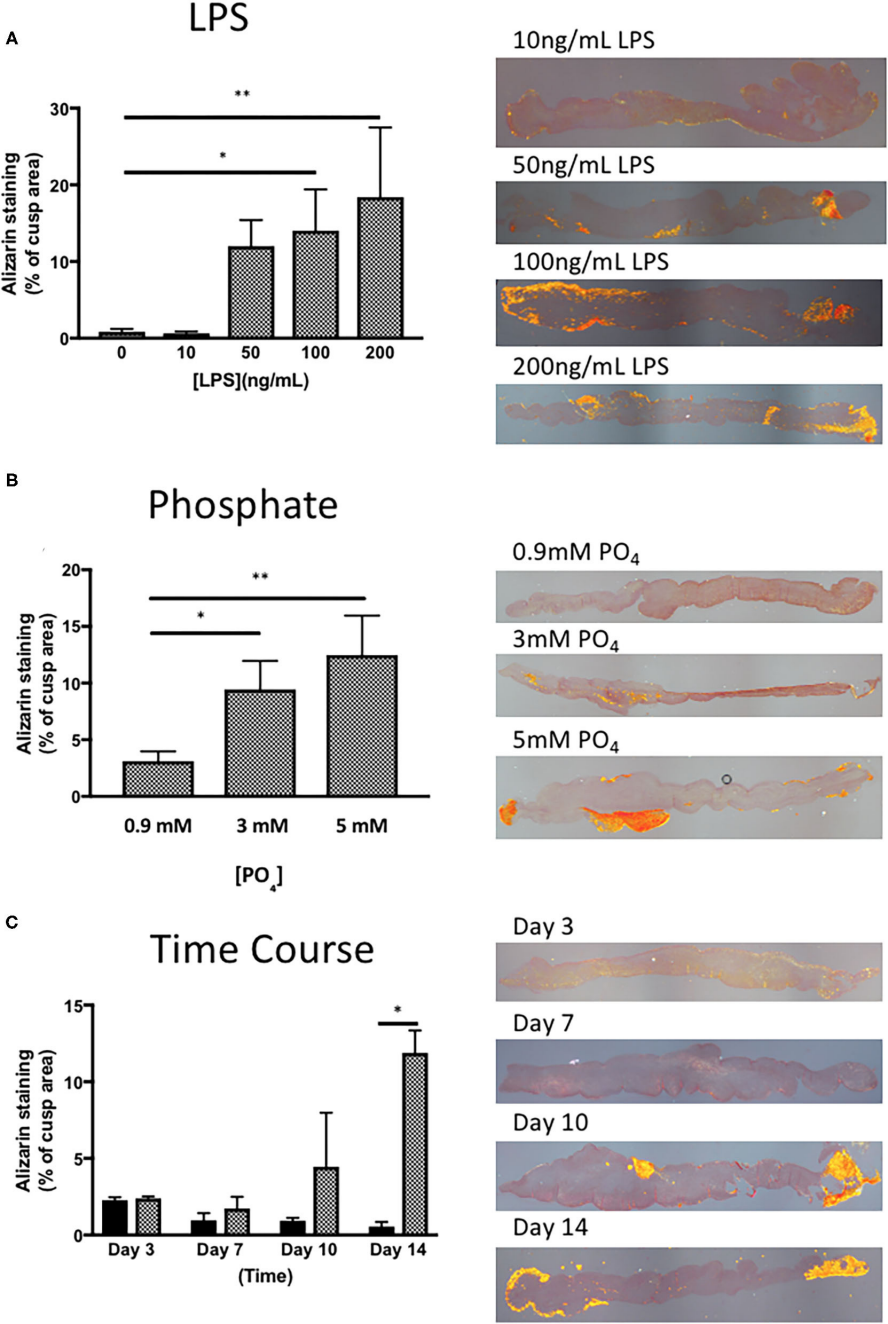
Optimisation of the response of valve leaflets to LPS and phosphate. **(A)** The effect of LPS concentration on calcium accumulation in porcine aortic valve leaflets in culture for 14 days in the presence of 3 mM phosphate (*p = 0.071, **p = 0.003, ANOVA; *n* = 3) and representative images of sections stained with Alizarin Red, imaged under polarized light for each concentration of LPS. **(B)** The effect of phosphate concentration on calcium accumulation in porcine aortic valve leaflets in culture for 14 days in the presence of 100 ng/mL LPS (*P = 0.015, **P = 0.001, ANOVA; *n* = 4) and representative images of sections stained with Alizarin Red, imaged under polarized light for each concentration of phosphate. **(C)** Time course of the effect of culture media (black bars) and 100 ng/mL LPS and 3 mM phosphate (cross-hatched bars) on calcium accumulation in porcine aortic valve leaflets in culture for 3 to 14 days (*P = 0.001, 2 way ANOVA, *n* = 2) and representative images of sections stained with Alizarin Red, imaged under polarized light for each time point.

To identify a suitable concentration of LPS to use, the effect of 10–200 ng/mL of LPS was assessed in the presence of 3 mM phosphate for 14 days. LPS was able to give a concentration-dependent increase in Alizarin Red staining in porcine valve leaflets, where the response of LPS at 10 ng/mL was indistinguishable from the control group (no LPS), while that of 100 & 200 ng/mL showed that 15–20% of the cross-sectional area of the leaflet stained positive for Alizarin Red (Figure 2A). In another series of experiments, to optimize the concentration of inorganic phosphate, the concentration of LPS was kept at 100 ng/mL and the concentration varied of phosphate between 0.9 mM and 5 mM (DMEM contains 0.9 mM phosphate) for 14 days. Supplementation of DMEM with 3 mM and 5 mM phosphate gave a progressive increase Alizarin Red staining up to ~20% of the valve area (Figure 2B). Based on the findings of these initial experiments, all subsequent experiment used 100 ng/mL of LPS and 3 mM phosphate. To check if the incubation time of LPS and phosphate was optimal, a time-course experiment over the 14-day period was performed. After 3 and 7-days incubation with 100 ng/mL LPS and 3 mM phosphate, there were no measurable increases in Alizarin Red staining. After 10 days there was marked, but variable increase in calcification, which was further increased after 14-days (Figure 2C). These results confirmed that the experiments required at least 14 days of incubation with LPS and phosphate.

### Leaflet Specific and Regional Effect of Calcification Response

**Figure 3:**
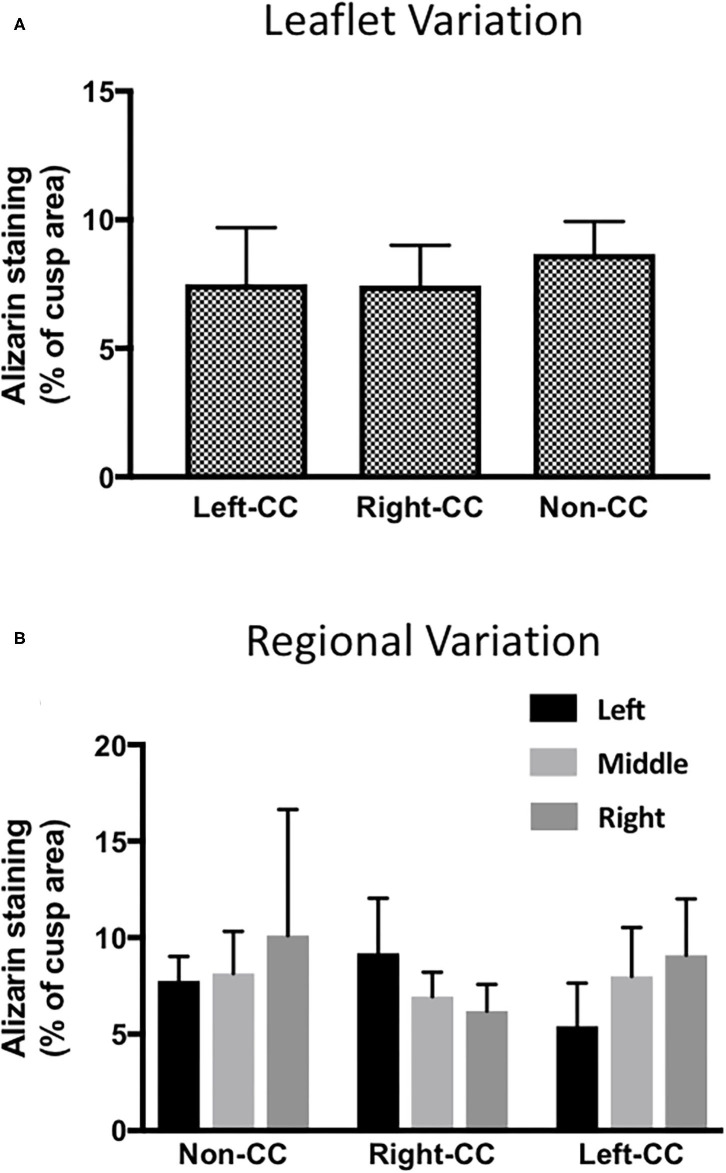
Leaflet-specific and regional effects of LPS and phosphate. **(A)** Leaflet specific effects of 100 ng/mL LPS and 3 mM phosphate on calcium accumulation in the left-, right- and non-coronary leaflets of porcine aortic valves. Values are the mean of the measurement from the three regions of each leaflet (*p* > 0.05, ANOVA; *n* = 6). **(B)** Comparison of the effect of 100 ng/mL LPS and 3 mM in the 3 regions of the left- right- and non-coronary leaflet assessed for calcium accumulation (*p* > 0.05, ANOVA; *n* = 6).

To check if there was any variation in the calcification response of the left-coronary, right-coronary and non-coronary leaflets, we analyzed the response of each individual leaflet to 100 ng/mL LPS and 3 mM phosphate. Results show that the calcification response did not differ between the three different valve leaflets (Figure 3A). To validate the measurement of calcification in the three regions across each leaflet, the response in the left, middle and right portion of each leaflet, in each of the three leaflets was compared. No statistical difference in the response of each region, in each cusp could be seen (Figure 3B). It was noteworthy that the right portion of the non-coronary cusp was markedly more variable than the other regions studied.

### EDAX Elemental Analysis

**Figure 4:**
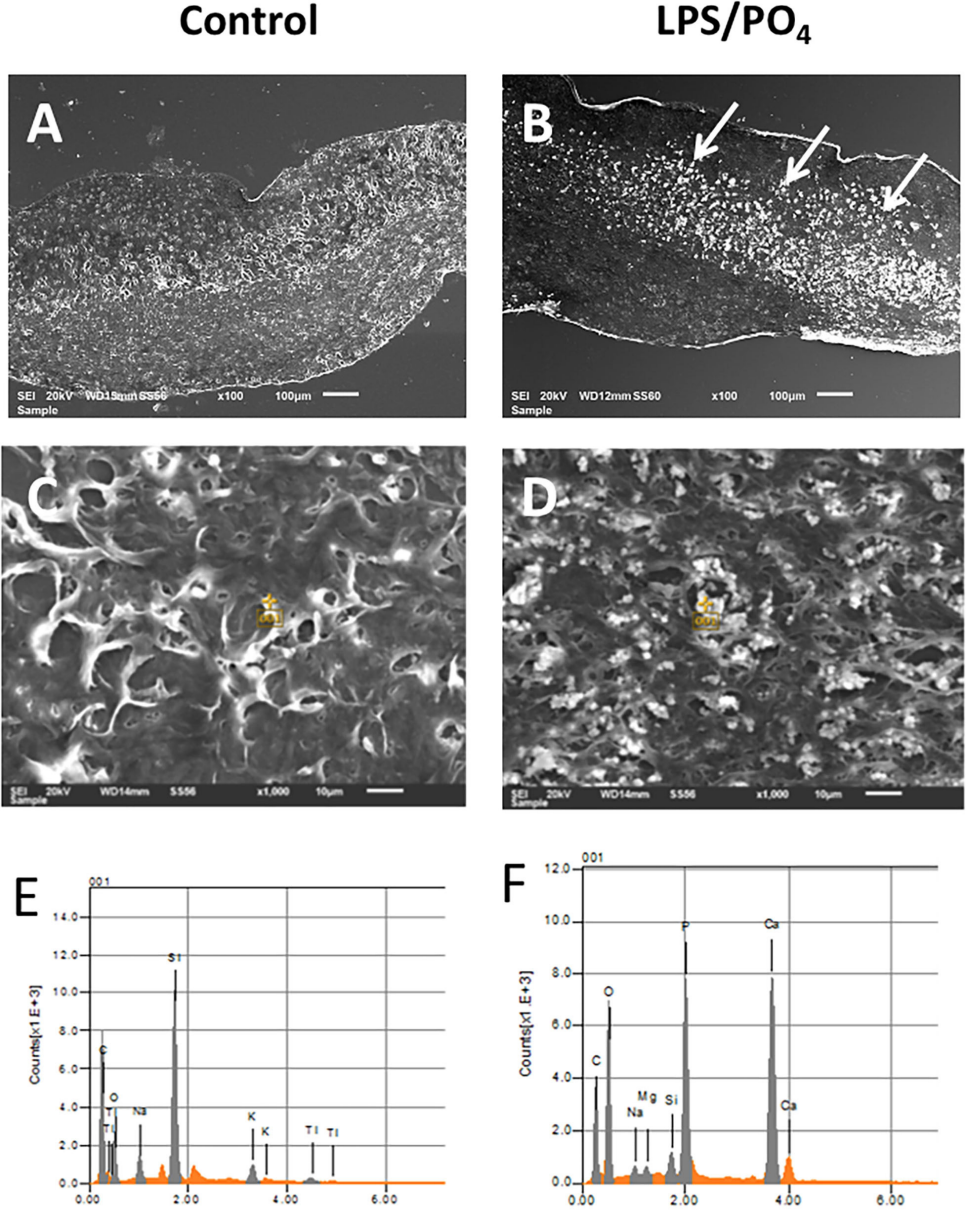
Electron microscopy and EDAX analysis of calcified valve leaflets. Scanning electron micrographs of porcine valve leaflets stimulated with media alone **(A)** or 100 ng/mL LPS and 3 mM phosphate for 14-days **(B)**, with presence of calcified areas (white arrows) (x100 magnification, scale bar = 100 μM). High power view and point analysis (yellow cross) of aortic valve leaflets from control and LPS/PO4 treated valves (x1,000 magnification, scale bar =10 μM) **(C,D)**, and the spectrum showing no calcium or phosphorus peaks in the control **(E)**, but their presence in the LPS/PO4 treated tissue **(F)**.

**Figure 5:**
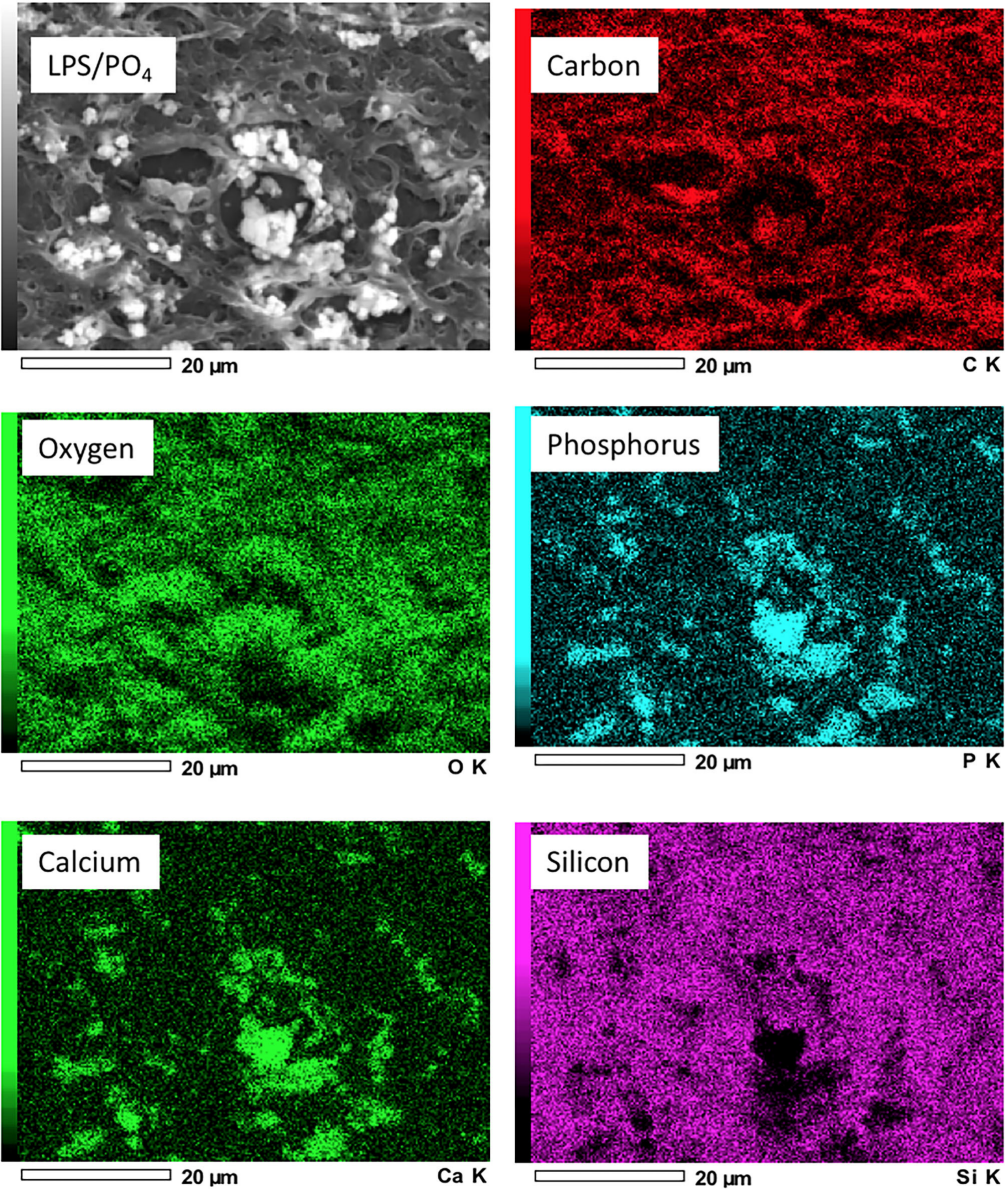
Elemental mapping of LPS and phosphate treated valve leaflets. Scanning electron micrographs showing the elemental distribution of carbon, oxygen, phosphorus, calcium and silicon. Regions of greater electron density corresponding to the distribution of calcium and phosphorus.

Scanning electron microscope images of valve treated with 100 ng/mL LPS and 3 mM phosphate showed electron dense nodules in specific regions of the leaflet, which were not evident in the media only treated tissue (Figures 4A,B). Point analysis of LPS/phosphate stimulated valve leaflets by EDAX showed a prominent peak in the spectra for calcium at 3.69 KeV and for phosphorus at 2.01 KeV. In contrast, media treated valve leaflets showed no peak corresponding to calcium or phosphorus. The appearance of silicon in the spectra is due to inadvertent detection of the glass slide (Figures 4C–F). In addition to the spectral analysis, the chemical nature of the image seen on the electron micrographs was confirmed with elemental mapping, whereby the presence of calcium and phosphorus coincided with the image of the nodule (Figure 5).

### Expression of Inflammatory, Calcification and Apoptotic Markers by Immunohistochemistry

**Figure 6:**
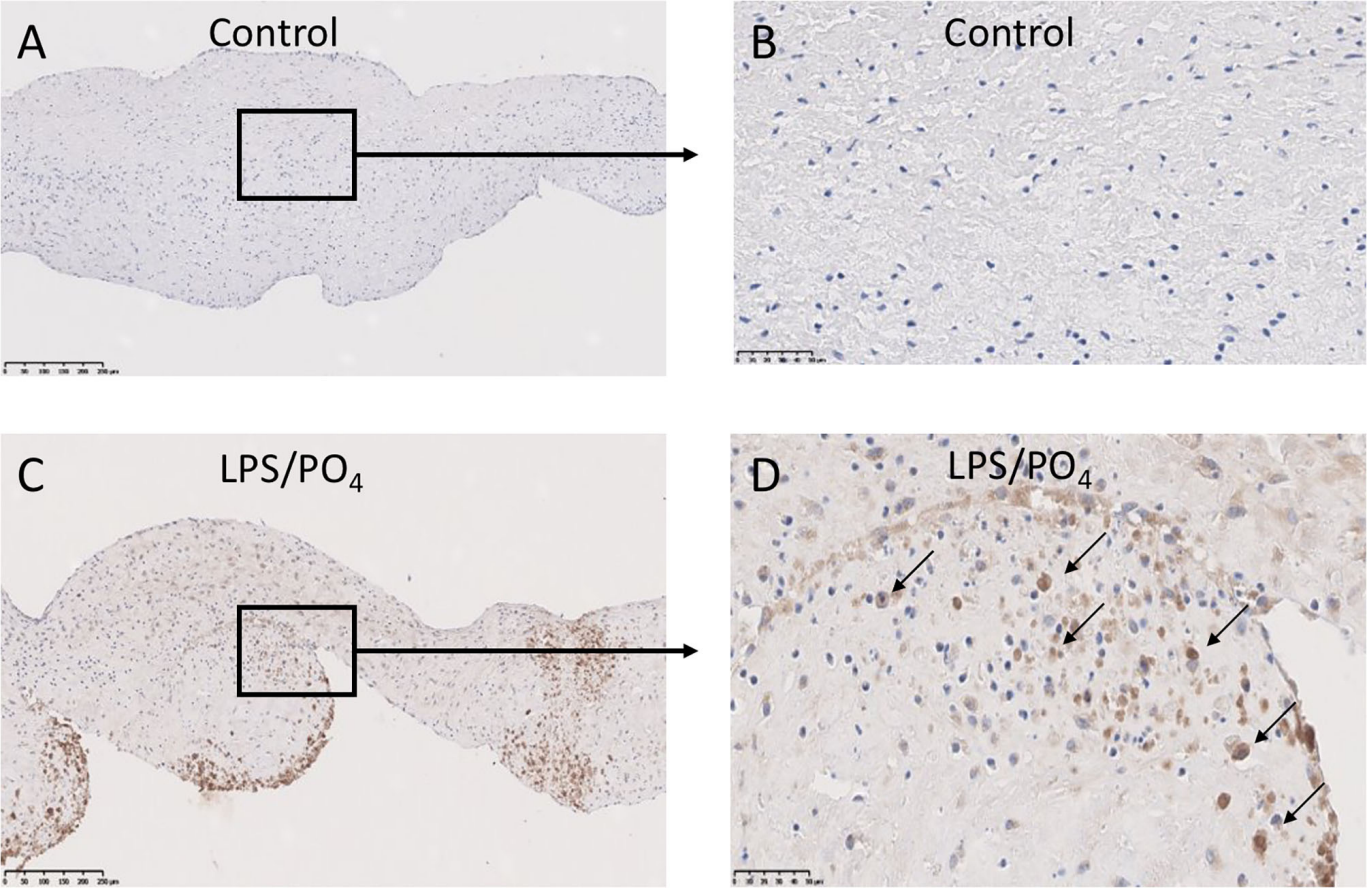
Expression of NF-kB in calcified valve leaflets. Low and high-powered images of immunohistochemical staining for NF-kB in untreated **(A,B)** and 100 ng/mL LPS & 3 mM phosphate treated **(C,D)** valve leaflets. Nuclear expression of NF-kB can be seen in **(D)** (black arrows). **(A,C)** scale bar, 200 μM; **(B,D)** scale bar, 500 μM.

**Figure 7:**
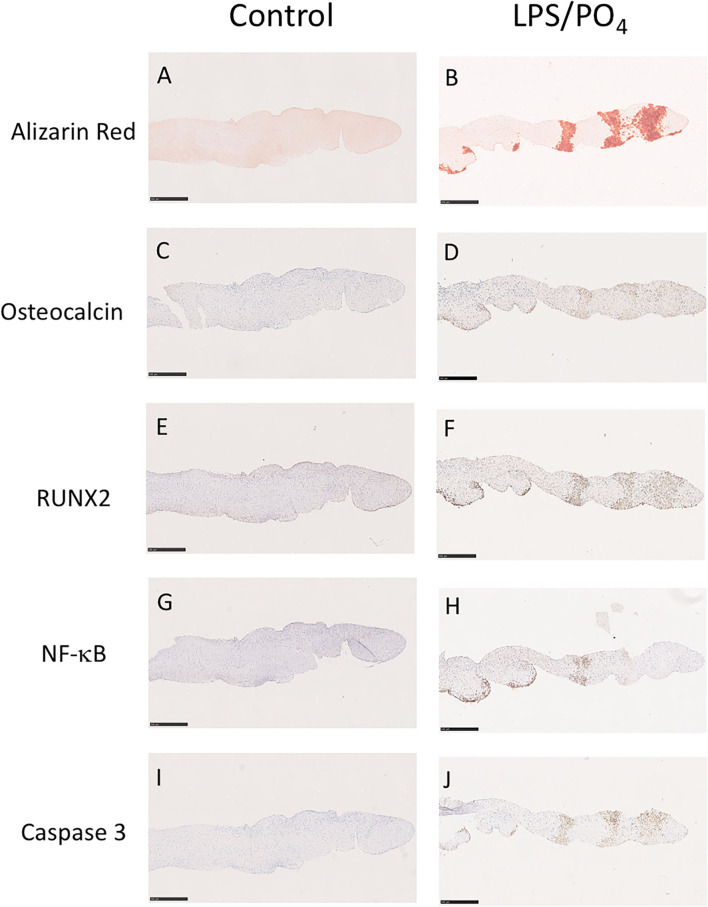
Colocalisation of Alizarin Red staining with osteogenic, inflammatory and apoptotic markers. Sequential sections of a valve leaflet stimulated media alone (left column) or 100 ng/mL LPS and 3 mM phosphate (right column) for 14-days. Histochemical staining for Alizarin Red **(A,B)** co-localizes with regions of immunohistochemical staining for osteocalcin **(C,D)**, RUNX2 **(E,F)**, NF-kB **(G,H)** and caspase 3 **(I,J)**. Scale bar, 500 μM.

**Figure 8:**
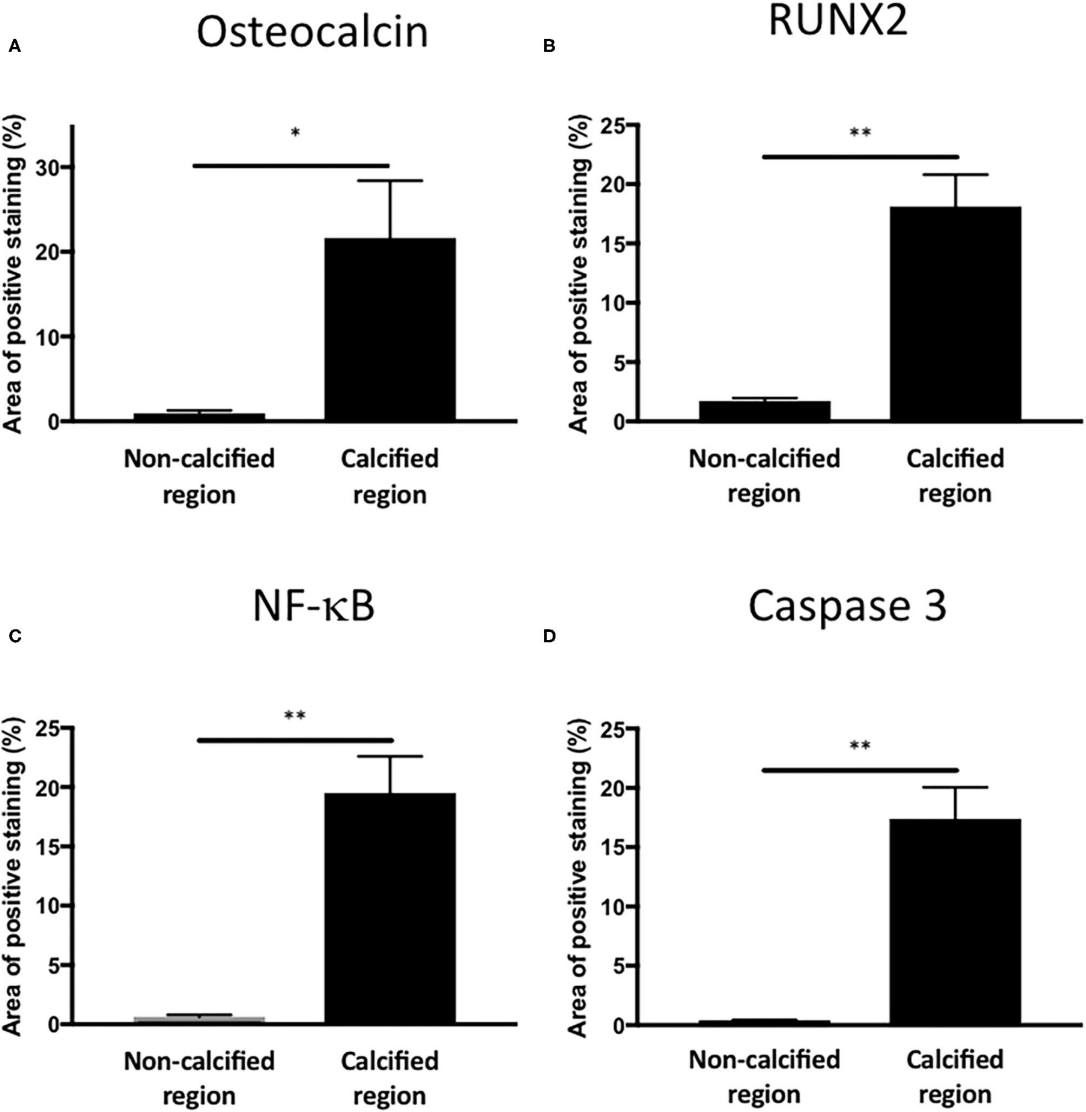
Quantification of osteogenic, inflammatory and apoptotic markers in Alizarin Red positive and negative areas of valve leaflets. Percentage area staining of 1.1 mm^2^ regions from non-calcified and calcified regions for **(A)** osteocalcin (*P = 0.012, *T*-Test; *n* = 4), **(B)** RUNX2 (**P < 0.001, *T*-Test; *n* = 4), **(C)** NF-kB (**P < 0.001, *T*-Test; *n* = 4) and **(D)** caspase 3 (**P < 0.001, *T*-Test; *n* = 4).

To assess if LPS was inducing an inflammatory response in the valve leaflet, the expression of NF-kB was investigated. In untreated leaflets no staining for NF-kB was evident (Figures 6A,B). In contrast LPS and phosphate treated leaflets show regional staining, some of which that associated with the cell nuclei (Figures 6C,D). By examining the staining in sequential sections of valve leaflets from media treated (Figures 7A,C,E,G,I) and LPS and phosphate (Figures 7B,D,F,H,J), it was possible to show that areas that showed positive staining for Alizarin Red (Figure 7B) were associated with areas of positive staining for osteocalcin, RUNX2, NF-kB and caspase 3 (Figures 7D,F,H,J, respectively). Quantification of the staining in Alizarin Red positive areas, compared to areas that were negative for Alizarin Red, show that there were significantly greater levels of staining for osteocalcin, RUNX2, NF-kB and caspase 3 in the calcified areas (Figures 8A–D, respectively). The lack of caspase 3 staining in control valves indicated the absence of apoptosis after 14-days incubation in media alone.

### Pharmacological Modulation of Porcine Leaflet Calcification

**Figure 9:**
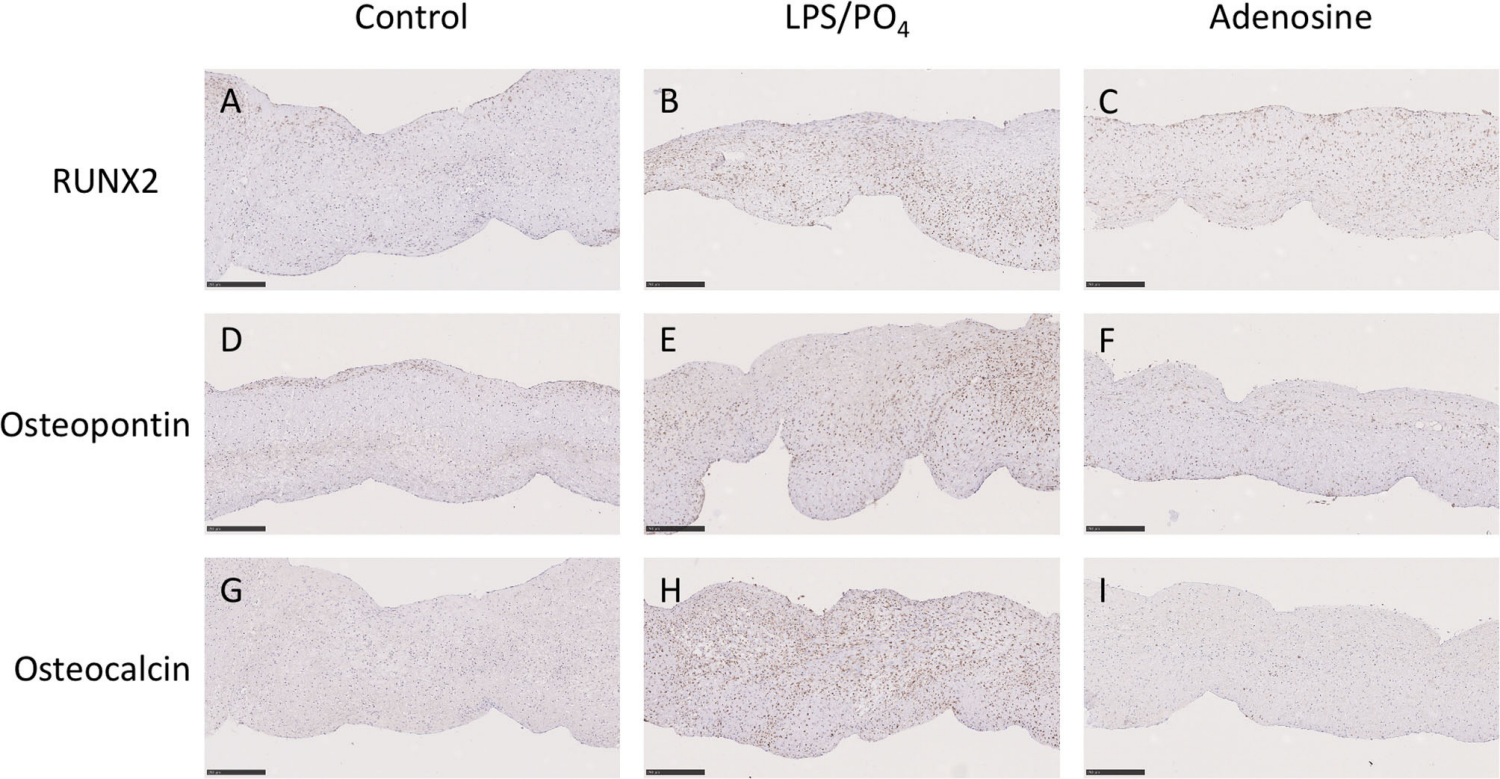
Effect of adenosine on the expression of osteogenic markers. Immunohistochemical staining in media alone (left column), 100 ng/mL LPS 3 μM phosphate treated (center column) and 100 ng/mL LPS 3 mM phosphate and 10^−5^M adenosine treated (right column) valve leaflets. Section were stained with RUNX2 **(A–C)**, osteopontin **(D–F)** and osteocalcin **(G–I)**. Scale bar, 100 μM.

**Figure 10:**
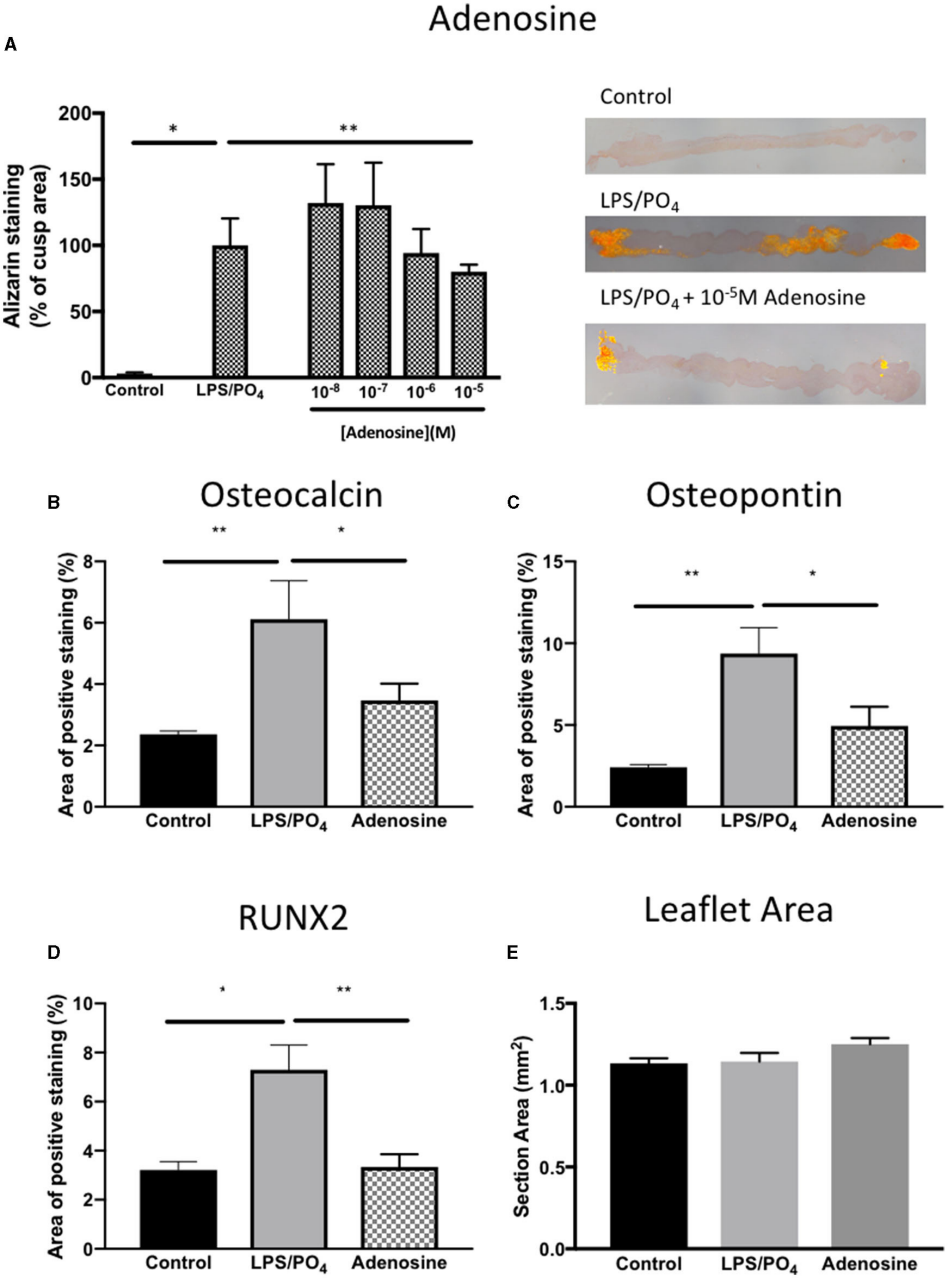
Inhibitory effect of adenosine on calcification and expression of osteogenic markers. Effect of increasing concentrations of adenosine (10^−8^-10^−5^M) on the expression of Alizarin Red in sections of valve leaflet response to 100 ng/mL and 3 mM phosphate (*P = 0.008, *T*-Test; *n* = 8 and **P = 0.011, *T*-Test; *n* = 6) and representative images of sections stained with Alizarin Red imaged under polarized light for each treatment group **(A)**. Quantification of the area of positive staining in control (media alone), 100 ng/mL and 3 mM phosphate and 100 ng/mL & 3 mM phosphate with 10^−5^M adenosine for **(B)** osteocalcin (**P = 0.001, *P = 0.029, ANOVA; *n* = 11–15 areas from 3 valves), **(C)** osteopontin (**P < 0.001, *P = 0.029, ANOVA; *n* = 13–15 areas from 3 valves) and **(D)** RUNX2 (*P = 0.003, **P = 0.001, ANOVA; *n* = 9–14 areas from 3 valves). **(E)** The mean area per measurement was similar in all 3 groups.

Based on our previous observations, where adenosine was shown to inhibit the expression of osteoblast marker in cultured human VIC (10), we assessed the ability of adenosine to inhibit the calcification of intact porcine leaflets stimulated with 100 ng/mL of LPS and 3 mM phosphate for 14 days. In these experiments, one leaflet from each valve was used as a positive control, while the other two leaflets were treated with adenosine. The effects observed in the adenosine treated leaflets were expressed as a percentage of the positive control for the corresponding valve. Immunohistochemical staining for RUNX2 (Figures 9A–C), osteopontin (Figures 9D–F) and osteocalcin (Figures 9G–I) showed stronger staining in the LPS and phosphate treated leaflets, compared to control. Addition of 10^−5^M adenosine to LPS and phosphate, resulted in a marked reduction in the staining for RUNX2, osteopontin and osteocalcin. Lower concentrations of adenosine (10^−8^ and 10^−7^M) had no significant effect on the amount of Alizarin Red staining in the leaflets. At concentrations above 10^−7^M, there was a progressive reduction in the amount of Alizarin Red staining, which reached statistical significance at 10^−5^M (Figure 10A). Corresponding to reductions in Alizarin Red staining, there was also significant reductions in antibody staining for osteocalcin, oeteopontin and RUNX2 in valve leaflets treated with 10^−5^M adenosine (Figures 10B–D, respectively). The areas measured for staining of each antibody were similar in all 3 groups (Figure 10E).

## Discussion

This study sets out an *ex-vivo* organ culture model of valve calcification that can quantify the level of calcium incorporated into the valve and assess phenotypic changes in the cells that reside in calcified regions. Elemental analysis and mapping showed that the nodules that were visible with the scanning electron microscope were comprised of calcium and phosphorus. The use of intact valve leaflets has the advantage that the VIC are retained in the physiological arrangement with respect to their association with the extracellular matrix and their relationship with the VEC. Intact valve leaflets from pigs and mice have previously been used to assess the effects of pro-calcific stimuli, including inorganic phosphate osteogenic media, osteogenic media supplemented with TGFβ1 and mechanical injury (11, 12, 21–24).

These previous studies have largely relied upon osteogenic media, which contains β-glycerophosphate, dexamethasone, and ascorbic acid, to drive the calcification response. In contrast, this model uses LPS, a TLR4 receptor agonist, to drive the calcification response and the subsequent initiation of an inflammatory response, as evidenced by nuclear expression of NF-kB in the calcified regions. The contribution of specific cell types within the valve leaflet to the response of LPS was not studied, however LPS has previously been shown to stimulate a calcification response in cultured VIC and to augment the effects of calcification of VIC to gamma–interferon (18, 20, 25, 26). Phosphate in combination with inorganic pyrophosphatase has also previously been shown to stimulate increase levels of radiolabeled calcium and the formation of Alizarin Red positive lesions, in intact valve leaflets over an 8-day period (12). Based on these findings we included pyrophosphatase to block the anti-calcification effect of pyrophosphate in the valve leaflets. We observed an augmentation of the response to 100 ng/mL LPS, with increasing phosphate concentration. Since there was no significant difference in the effect between 3 and 5 mM phosphate, we opted to choose the lower concentration, since this is nearer to the concentration used in previous studies and below the concentration required for phosphate to have a direct effect on calcification (12, 14).

From the analysis of the data on the quantification of the percentage area of each leaflet that showed positive staining with Alizarin Red, we determined that the most accurate way to assess the degree of calcification in each valve leaflet was to sample each leaflet in three areas and calculate the mean of the three observations. We were subsequently able to show that there was no bias toward any of the three individual aortic valve leaflets or to left, middle or right regions of each of the cusps. The Alizarin Red staining did not follow a specific pattern, nor was it exclusively associated with the fibrosa layer of the valve leaflets. There was a tendency for the calcification to occur at either end of each cusp. This could be due to the development of tension in the cusp when it was pinned into position in the culture plate or during the 14-day culture period. We aim to extend this model with the use of a stretch bioreactor to investigate the application of cyclic strain on the calcification response of LPS and phosphate. It has been previously shown that strain increased calcified nodule formation and enhances the response to TGFβ1 and BMP (11, 27). The advantage offered by this model includes the ability to quantify changes in cell phenotype and of calcification and adjacent sections of valve leaflets. There was increased expression of osteoblast markers and the osteoblast transcription factor RUNX2, both of which are known to be expressed in calcified valves (28, 29). The presence of caspase-3 in the calcified regions demonstrates a role for apoptosis in the calcification response, suggesting that dystrophic calcification may also be occurring alongside osteogenic calcification. Both osteogenic and dystrophic calcification are features of the disease in human valves (30, 31). This model gives the opportunity to probe for other markers or mediators of calcification via antibody staining and/or western blotting. Recently a multi-omics approach to define the pathogenesis of calcific aortic valve disease has been advocated (32). Comparing the changes induced in this model using transcriptomics, proteomics and metabolomics will define the pathways that mediate the response and allow comparison with cell-based models and pathological samples from humans.

We were also able to show that the calcification response was amenable to pharmacological manipulation with adenosine. Our previous studies have shown an inhibitory effect of osteogenic markers induced by osteogenic media (10). Here we were able to demonstrate an inhibitory effect of adenosine on levels of calcification induced by LPS and phosphate, which was associated with a reduction in osteopontin, osteocalcin and RUNX2 expression. The beneficial effects on adenosine on valve calcification have previously been reported in a murine aortic root challenged with osteogenic media. A_1_ and A_2b_ receptors were shown to mediate anti-calcification effects of adenosine, while A_2a_ receptor exacerbated the calcifying effect of osteogenic media (33). The model described in this paper would be amenable to similar receptor antagonist studies.

There are several limitations of the model described in this paper. The effect of LPS and phosphate takes at least 2 weeks to develop. A significant degree of calcification was observed after 10 days in some valves, but overall, the effect was more consistent with a longer incubation time. The data for time-course experiment in Figure 2C was based data from only 2 animals. While this experiment was only intended only to provide evidence of the shortest incubation time to give consistent response to LPS and inorganic phosphate, further studies with more samples and longer time points may demonstrate the need for a longer duration for the experiment to give less variation in the response of the leaflets. By keeping each valve leaflet intact, a larger number of hearts are required to run more complex experimental protocols (e.g., concentration-response, antagonist studies). We avoided cutting each valve into smaller pieces, to avoid damage to the endothelial layer and maintain the integrity of the valve structure. Lastly, this model relies on the use of porcine tissue, which may not respond in an identical manner to human tissue. However, this type of study with human tissue would be virtually impossible due to the number of valves that would be required.

This organ culture model relies upon a combination of a biological stimulus and the promoting effects of phosphate to yield a quantifiable calcification response and an opportunity to simultaneously identify changes in the expression of phenotypic markers associated with valve calcification. We demonstrate that the model is amenable to pharmacological modulation by adenosine. The presence of all the cellular components and extracellular matrix will allow future studies to identify new markers and mediators of valve calcification as well as to serve as a tool for pre-clinical assessment of new anti-calcification agents.

## Data Availability Statement

The raw data supporting the conclusions of this article will be made available by the authors, without undue reservation.

## Ethics Statement

Ethical review and approval was not required for the animal study because use of porcine hearts obtained from abattoir being slaughtered for food.

## Author Contributions

AC: devised concept and experimental design, analysis of data, and writing of the paper. PS: histology, EM work, elemental analysis, and writing of the paper. AM: valve tissue preparation and culture, processing samples, and writing of the paper. MY: critical appraisal and writing of the paper. All authors contributed to the article and approved the submitted version.

## Funding

This study was funded by The Magdi Yacoub Institute.

## Conflict of Interest

The authors declare that the research was conducted in the absence of any commercial or financial relationships that could be construed as a potential conflict of interest.

## Publisher's Note

All claims expressed in this article are solely those of the authors and do not necessarily represent those of their affiliated organizations, or those of the publisher, the editors and the reviewers. Any product that may be evaluated in this article, or claim that may be made by its manufacturer, is not guaranteed or endorsed by the publisher.
